# Enrichment of Quercetin from Winemaking Residual Diatomaceous Earth via a Tailor-Made Imprinted Adsorbent

**DOI:** 10.3390/molecules27196406

**Published:** 2022-09-28

**Authors:** Amir Bzainia, Rolando C. S. Dias, Mário Rui P. F. N. Costa

**Affiliations:** 1 Polytechnic Institute of Bragança, Mountain Research Center (CIMO), 5300-253 Bragança, Portugal; 2 LSRE-LCM-Laboratory of Separation and Reaction Engineering—Laboratory of Catalysis and Materials, Faculty of Engineering, Department of Chemical Engineering, University of Porto, 4200-465 Porto, Portugal

**Keywords:** residual diatomaceous earth, molecularly imprinted polymers, quercetin, enrichment

## Abstract

Residual diatomaceous earth (RDE) from winemaking activities is a rich and currently underexploited source of phenolic compounds which ought to be recycled from the perspective of circular bioeconomy. In this work, we demonstrate the feasibility of molecularly imprinted polymers (MIPs) for the enrichment of quercetin, a flavonoid at a fairly high content in residual diatomaceous earth. These MIPs were synthesized through free radical polymerization. FTIR confirmed the integration of the functional monomers into the polymeric chains. Batch adsorption experiments were used to assess the retention and selectivity of those MIPs towards quercetin. Commercial resins were compared with the synthesized materials using the same procedures. These adsorption experiments allowed the selection of the best performing MIP for the valorization of RDE extract. This treatment consisted of saturating the selected MIP with the extract and then desorbing the retained compounds using solvents of selected compositions. The desorbed fractions were analyzed using liquid chromatography, and the results demonstrated an increase in quercetin’s fractional area from 5% in the RDE extract to more than 40% in some fractions, which is roughly an eightfold enrichment of quercetin. Moreover, other flavonoids of close chemical structure to quercetin have been rather retained and enriched by the MIP.

## 1. Introduction

Wine production has been carried out for the last 8000 years, since the Neolithic period [1], and nowadays, it is a preponderant agro-industrial activity in Europe with a production level reaching 159 million hl in 2020 [2]. Needless to say that a sector of this prominence has its own drawbacks which manifest in the generation of large amounts of solid and liquid wastes [3]. The latter consists of the effluent wastewater [4], whereas the former comprises grape pomace, vine shoots, grape stalks, wine lees and wine filter waste [5]. The most common filtering medium is diatomaceous earth which is mostly an amorphous silica network of a high specific surface area arising from micro- and nanopores [6,7].

These different winemaking residues are rich in phenolic compounds which are highly praised for their antioxidant and anti-inflammatory properties able to alleviate neurodegenerative and cardiovascular diseases, thus making them very useful in the pharmaceutical, cosmetic and food industries [8,9,10,11]. For instance, administrating quercetin into rats improved their learning and memory capabilities as well as prevented neural cell apoptosis [12]. Another study showed that the flavonoid kaempferol inhibits the proliferation of breast cancer cells in persons previously exposed to the antimicrobial agent triclosan [13].

In addition, other industries such as packaging [14,15] and textile [16] ones are incorporating phenolic compounds into their products. In fact, caffeic acid and gallic acid were introduced in a packaging material to prevent the proliferation of bacteria [15]. Moreover, anthocyanins, which are responsible for the pigmentation of plants, are nowadays being sought by the textile industries to substitute synthetic dyes, thus decreasing the risk of contact allergies [16]. 

Based on these numerous applications, it is of great interest to extract and enrich these phenolic compounds to facilitate their use in the aforesaid industries. For this purpose, several techniques of enrichment are available such as membrane processing, which separates the phenolic compounds according to their effective molecular size [17]. In fact, this technology was applied to wine lees for the separation of the phenolic compounds from polysaccharides [18]. 

In addition, an emerging separation technique based on colloidal gas aphrons was also employed to separate phenolic compounds from sugars and proteins [19,20]. This technique relies on a fast-stirred surfactant solution generating microbubbles able to adsorb molecules due to charge attraction [19,20]. Additionally, the enrichment of polyphenols from olive oil wastewater was attempted using ionic and non-ionic resins showing an enrichment factor of twofold for the non-ionic resins [21]. 

The common disadvantage of the mentioned techniques is their poor selectivity, which poses a serious problem if the enrichment of a particular polyphenol is intended. To circumvent this issue, tailor-made adsorbents known as “molecularly imprinted polymers” (MIPs) can be brought into play. MIPs are synthetic materials capable of recognizing and binding to a precise molecular structure. Such selectivity is due to the specific cavities embedded in the polymeric network of the material that are created during the polymerization process [22,23,24,25,26]. Owing to their versatility and selectivity, MIPs have been used in various applications, namely as drug delivery vehicles, gas and liquid sensors, and in chromatographic separation processes [22,24,25,26]. Furthermore, MIPs have been employed as adsorbents in a continuous separation process to treat olive oil, wine, and chestnut residues. This allowed the enrichment of a plethora of phenolic compounds [23]. 

In this work, the synthesis of quercetin-imprinted polymers is described, and their adsorption capabilities are assessed in terms of selectivity and retention towards quercetin. This permitted discerning the MIP with the highest retention and selectivity towards quercetin. The chosen MIP was employed to treat residual diatomaceous earth (RDE) extract in a process of adsorption/desorption using solid phase extractor (SPE) apparatus. This MIP demonstrated an ability to recognize and bind to quercetin from an extract containing various bioactive compounds leading to the obtention of quercetin-rich fractions which are depleted of other interfering compounds.

## 2. Results and Discussion

### 2.1. Rationale for MIP Synthesis

The synthesis of a MIP should be preceded by a thorough study of the target molecule(s) as well as the conditions of the utilization of the material. The objective is to create a polymer capable of capturing a specific compound (or a fragment of that compound) in a selective way. This is achieved by polymerizing one or more functional monomers(s) (FMs) in the presence of the target molecule and a crosslinker leading to the creation of cavities with high affinity for the said compound. During the process of molecular imprinting, it is mandatory to preserve the interactions susceptible to occur between the FM(s) and the target compound. For instance, the use of solvents such as acetonitrile (ACN) or *N*,*N*-dimethylformamide (DMF) promotes hydrogen bond interactions, whereas protic solvents such as water would immensely tamper with the hydrogen bonds between the FM(s) and the target molecule. Ideally, one could choose the most suitable solvent to strengthen as much as possible the interactions between the FM(s) and the target molecule. However, the solubility of the different reagents (monomers, crosslinker, and initiator) and the target polyphenol could drastically change the “ideal” conditions for molecular imprinting. In the polymerizations performed herein, mixtures of ACN and DMF were used as synthesis solvents to fully dissolve the needed amounts of quercetin. 

Regarding the FMs, they were chosen based on the chemical structure of quercetin (target molecule). This flavonoid contains three aromatic rings, five hydroxyl functions and a carbonyl function which are all moieties susceptible to interacting with FM(s). Therefore, three different FMs were chosen to synthesize the MIPs: 4-vinylpyridine (4VP), styrene (STY) and 1-(4-vinylphenyl)-3-(3,5-bis(trifluoromethyl)phenyl)-urea (4VAN-BTPI). This third FM is not commercially available and so it had to be synthesized in the framework of this research.

**Figure 1:**
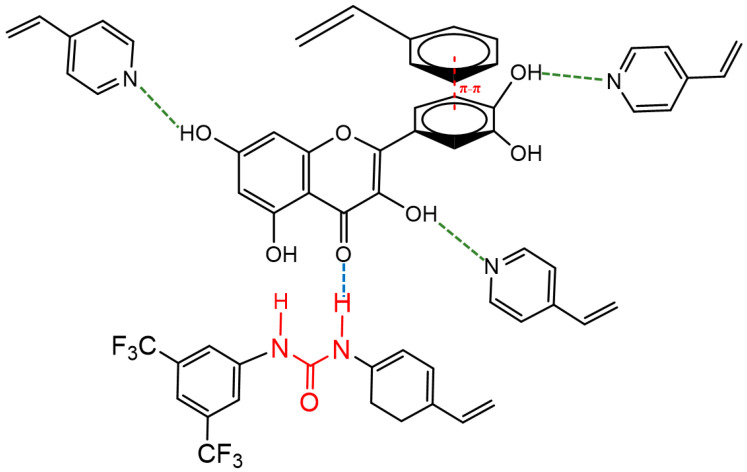
Predicted interactions between quercetin and FMs.

The predicted interactions of quercetin with the FMs are depicted in Figure 1. 4VP establishes hydrogen bonds with the hydroxyl functions, whereas STY interacts through π-π stacking with the aromatic rings of quercetin. Finally, due to the urea moiety, 4VAN-BTPI could interact with the carbonyl function of quercetin.

Bearing in mind this reasoning, four different MIPs and their corresponding non-imprinted polymers (NIPs) were synthesized. Table 1 summarizes the conditions which were used in each synthesis (MQ refers to the MIP while NQ refers to the NIP). The parameters shown in the table are a way to describe the initial state of the polymerization system and they are defined as:

- Y_M_ is the mass fraction of FMs and the crosslinker in the solution (%);
- Y_I_ is the mole fraction of initiator relative to the FMs and the crosslinker (%);
- Y_CL_ is the mole fraction of the crosslinker in the mixture of FMs/crosslinker (%);
- Y_FM/T_ is the mole ratio of the FM to the template molecule.

Altering these parameters directly impacts the morphology as well as the retention/selectivity of the final MIP. In this work, Y_M_ was varied from 6% to over 23%, leading, respectively, either to microparticles or to bulky materials.

Furthermore, the use of a crosslinker is indispensable for the creation of the three-dimensional cavities of the MIPs. However, a too-high content of crosslinker could negatively influence the specificity of the MIP towards the target molecule. Hence, for MQ1/NQ1 and MQ2/NQ2, a Y_CL_ of 83.33% was chosen whereas for MQ3/NQ3 and MQ4/NQ4, a Y_CL_ of 40% was chosen to provide a higher overall functionality to the materials. 

Regarding the value of Y_FM/T,_ it should be ideally in concordance with the number of moieties of the target molecule. For example, if a compound has four hydroxyl functions, then using four 4VP would in theory increase the specificity of the MIP. However, this logic does not take into account the steric hindrance limiting the number of interacting functional monomers. So, in this work, the materials were synthesized using values of 2 and 1 for Y_FM1/T_ and Y_FM2/T_, respectively. Such values have also been reported in other studies [27,28].

The last parameter intervening in the MIP/NIPs’ synthesis is the amount of initiator. High values of Y_I_ can lead to short polymer chains since many molecules of monomers yield propagating radicals at the same time. Obviously, this is not desired because the MIP would not contain the sought cavities to bind the target molecule. On the other hand, using low amounts of initiator could not overcome the inhibition of the polymerization when using a polyphenol as a template (similarly to their antioxidant role). In the current study, values around 5% of Y_I_ were suitable for the polymerizations’ systems and led to high yields.

#### FTIR Characterization

**Figure 2:**
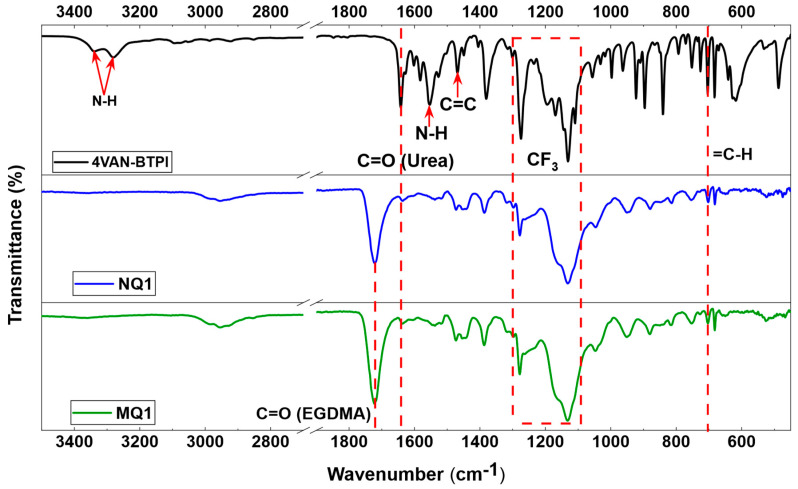
FTIR spectra of the 4VAN−BTPI monomer and its corresponding MIP and NIP.

Fourier-transform infrared spectroscopy (FTIR) is a straightforward analytical technique often providing accurate information about the final composition of the synthesized material. This technique was used to analyze the MIPs/NIPs synthesized in this work as well as the 4VAN-BTPI monomer. The FTIR spectrum of the latter as well as the derivative polymers is shown in Figure 2. The spectrum of the urea monomer (4VAN-BTPI) reveals characteristic peaks around 1642 cm^−1^, 1552 cm^−1^ and 1468 cm^−1^ which are assigned, respectively, to the C=O stretching of a secondary amide, the N-H deformation of a secondary amide, and the stretching of aromatic C=C attached to an electron-withdrawing group (CF_3_ in this case). Moreover, the absorbance peaks around 3340 cm^−1^ and 3280 cm^−1^ correspond to the symmetrical and asymmetrical stretching of N-H of a secondary amide. Finally, the peaks between 1100 cm^−1^ and 1300 cm^−1^ are assigned to the stretching of the C-F functions (within the RCF_3_). The FTIR spectra of the corresponding polymers (MQ1 and NQ1) clearly show the incorporation of the 4VAN-BTPI monomer as well as the crosslinker EGDMA (C=O) in the final polymeric network.

**Figure 3:**
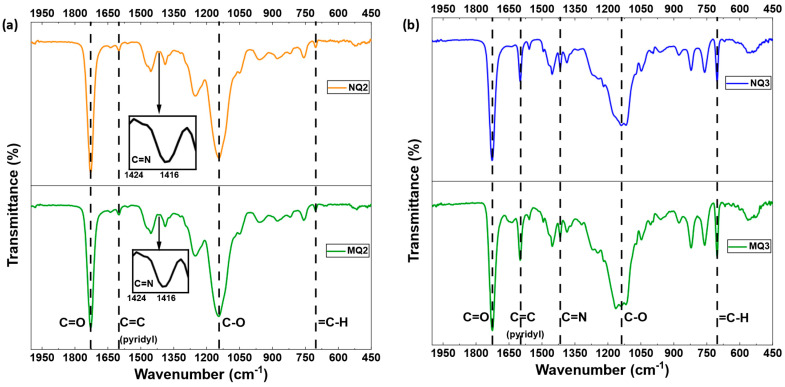
FTIR spectra of the 4VP−based MIPs. (**a**) MQ2/NQ2; (**b**) MQ3/NQ3.

Concerning the MIPs/NIPs synthesized with 4VP, their FTIR spectra are plotted in Figure 3 and Figure S1 (for MQ4 and NQ4). The peak at 1726 cm^−1^ is assigned to the stretching of C=O and the one at 1150 cm^−1^ is assigned to the stretching of an aliphatic ether C-O. Both peaks are indicators of the presence of the crosslinker EGDMA in the MIPs/NIPs. Furthermore, the peaks around 1600 cm^−1^ and 1415 cm^−1^ are assigned, respectively, to the stretching of the pyridyl C=C and the C=N functions that exist in the 4VP monomer. The peak at 702 cm^−1^ is typical of aromatic rings and indicates the presence of STY in the polymeric network. Overall, the FTIR spectra demonstrate that the FMs (4VP, STY and 4VAN-BTPI) and the crosslinker EGDMA had been successfully incorporated into the MIPs/NIPs.

### 2.2. Screening of the Potential Adsorbent for RDE Treatment

In order to enrich quercetin from RDE extract, batch adsorption tests were performed to screen the potential material able to selectively adsorb quercetin in a high amount. This screening step considered both synthetic materials (MIPs/NPs) and commercial resins.

**Figure 4:**
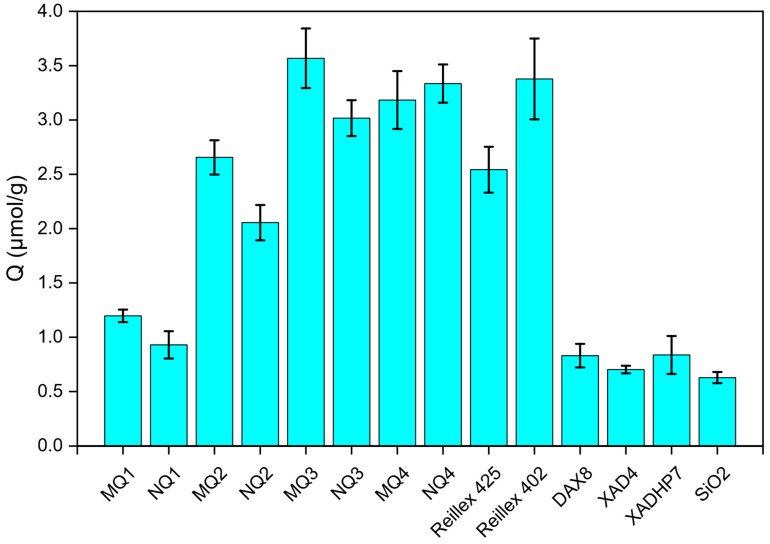
Batch sorption results in 8/2 EtOH/Water using 0.2 mM of quercetin.

In the first batch experiment, the synthesized materials (MIPs and corresponding NIPs) as well as the commercial resins (Reillex 402, Reillex 425, DAX8, XAD4, XADHP7 and silica-SiO_2_) were tested using a solution of quercetin. The results are displayed in Figure 4. At first glance, MQ3 showed the highest retention for quercetin as compared to the other MIPs/NIPs and the commercial resins. On the contrary, the materials made with 4VAN-BTPI showed a low adsorption capacity for quercetin. 

The higher retention of MQ3/NQ3 and MQ4/NQ4 as compared to MQ2/MQ2 is due to the high amount of crosslinker (EGDMA) in the polymeric network. In fact, as Y_CL_ increases, the number of FM units (4VP and STY) in the polymer chains decreases, whereas the number of crosslinker units increases. This translates into a lesser binding capacity of the material since the phenolic compound would not substantially interact with the FMs. 

Furthermore, MQ3/NQ3 supreme retention is explained by the polymer particle size which is determined by the parameter Y_M_. As this latter grows, the total concentration of EGDMA, 4VP and STY increases in the reaction mixture, resulting in the formation of polymers with higher particle size and, eventually, bulky materials. In case of MQ3/NQ3, Y_M_ was around 6%, thus yielding microparticles with a specific area larger than that of MQ4/NQ4 (YM = 21.76%), hence its significant retention of quercetin.

Among the synthesized materials, MQ1/NQ1, MQ2/NQ2 and MQ3/MQ3 demonstrated a molecular-imprinting effect towards quercetin with imprinting factors of 1.24, 1.43 and 1.22, respectively. In case of MQ4/NQ4, the imprinting factor was less than one, meaning that the material was not specific to quercetin in the conditions of analysis (EtOH/W 80/20). This can be attributed to the amount of DMF (30%) used in the solvent of synthesis which could weaken the interactions of the STY-quercetin and 4VP-quercetin, leading to a less specific MIP. Additionally, due to its inherent molecular structure, ACN interferes less with the hydrogen bonds than DMF does; thus, in a mixture of 85/15 ACN/DMF, quercetin would have stronger interactions with 4VP through hydrogen bonding than in a mixture of 70/30 ACN/DMF. 

Although MQ1/NQ1 had an imprinting factor higher than one, it had the lowest retention for quercetin compared to other synthetic materials. This is due to the scarce number of moieties on the quercetin where the urea monomer can bind (carbonyl function).

Comparing now with some commercial resins, Reillex 402 shows the highest retention for quercetin, which is an expected result since the material is made of 98% 4VP. Nonetheless, the adsorption capacity of Reillex 402 did not surpass that of MQ3 and both capacities were actually very close (3.90 and 3.96 µmol/g, respectively), which showcases the advantage of molecular imprinting in the targeting of quercetin. Reillex 425, which is made of 75% 4VP, also demonstrated a relatively high retention for quercetin but lesser than Reillex 402. DAX8 and XADHP7 are both acrylic resins [29] containing carbonyl functions in their structures, thus explaining their higher retention of quercetin as compared to silica and XAD4 (polystyrene-based resin) [29].

**Figure 5:**
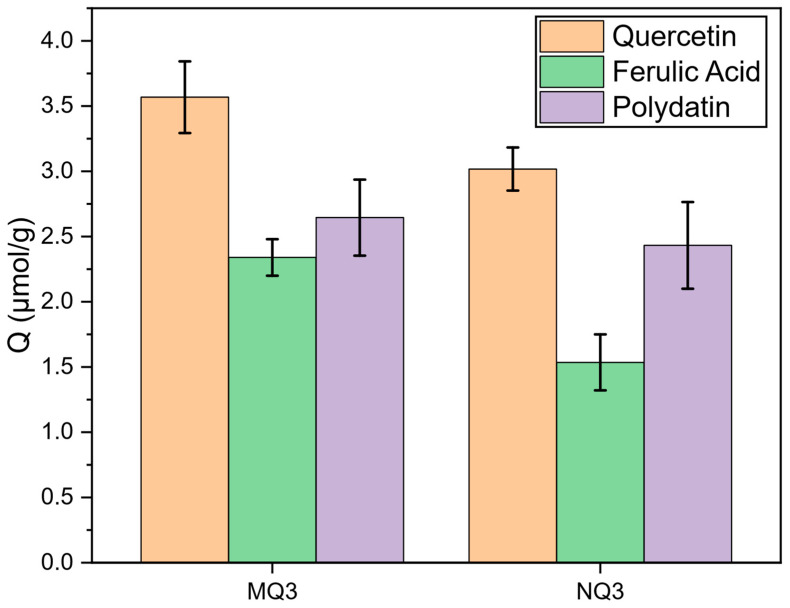
Batch adsorption results in 8/2 EtOH/Water using 0.2 mM of quercetin, polydatin or ferulic acid.

From the previous results, MQ3 showed the highest retention for quercetin which incited the investigation of its selectivity for the same compound. The separate adsorption experiments with ferulic acid (belonging to the phenolic acid family) and polydatin (belonging to the stilbene family) were carried out and the obtained results were compared to the quercetin batch adsorption as shown in Figure 5.

MQ3 exhibited a higher adsorption capacity for quercetin (3.96 µmol/g) than for ferulic acid (2.54 µmol/g) or polydatin (2.79 µmol/g) which evidences the selectivity of MQ3 towards quercetin. Interestingly, the adsorption capacity of MQ3 for ferulic acid and polydatin were slightly higher than NQ3, which is a feature that can be harnessed to fractionate the phenolic compounds present in the RDE extract.

To sum up, the screening batch adsorption tests demonstrated that MQ3 possesses the highest affinity for quercetin and, thus, it was chosen to be further used for the valorization of RDE extract in an attempt to enrich quercetin.

Moreover, it should be stressed that the molecular-imprinting effect and the selectivity of the materials are dependent on the used solvents which could weaken or strengthen the hydrogen bonding between the MIP and the phenolic compound(s). For instance, using protic solvents such as ethanol and water would decrease the strength of hydrogen bonding. On the other hand, other less H-bonding-competitive solvents such as acetonitrile would increase the observed imprinting effect (see references [23,30,31]). However, in the context of winemaking valorization for food, cosmetics, and pharmaceutical applications, we are avoiding the use of this kind of toxic solvent.

### 2.3. Residual Diatomaceous Earth Treatment with MQ3

**Figure 6:**
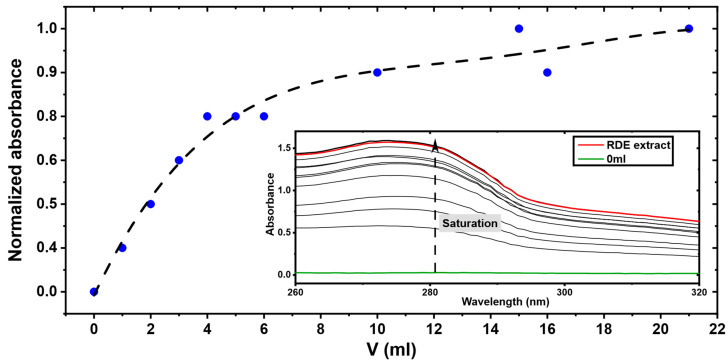
Breakthrough curve of MQ3 saturation. Inset: UV spectra of the SPE outlets during the saturation.

Using the SPE apparatus, the synthetic material MQ3 was saturated with RDE extract by passing 1 mL at a time. The saturation process was then stopped upon reaching the same UV absorbance of the RDE extract. Reaching the saturation is mandatory to ensure that the cavities of the polymeric network are all occupied which should allow a better evaluation of the adsorption capabilities of MQ3. Figure 6 shows the breakthrough curve obtained using RDE extract.

**Figure 7:**
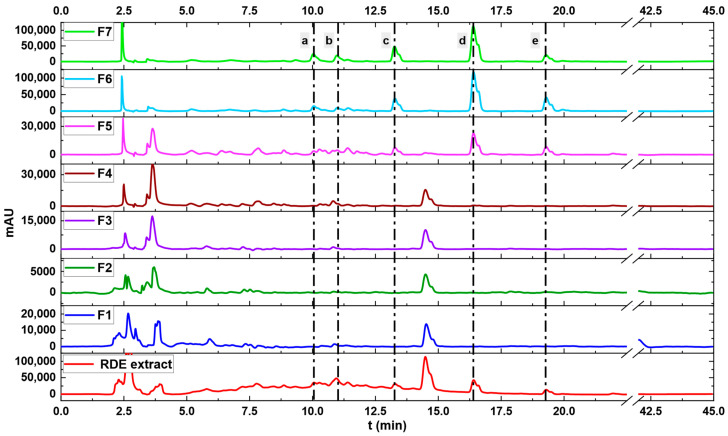
Fractions (F1 to F7) obtained after eluting the saturated MQ3 material.

The saturation step was followed by the fractionation one. The latter consisted of seven different elution solvents comprising pure water, mixtures of MeOH/Water, pure MeOH and, finally, MeOH/AcOH 90/10. Varying the eluting solvent allowed the obtention of fractions with a more simplified composition than the original RDE extract. The chromatograms of these fractions are presented in Figure 7.

As the primary interest of this paper is to evaluate the aptitude of a synthesized MIP to isolate/enrich quercetin from RDE extract, a standard of this phenolic compound was injected. Other compounds that eluted alongside quercetin were tentatively identified using the LC-MS-DAD data of the RDE. The results of this identification are summarized in Table 2. The peak around 14.8 min that appears in the chromatogram of the RDE extract is most likely a compound attributed to the vinification process as it did not correspond to any compound in the established library of polyphenols.

**Figure 8:**
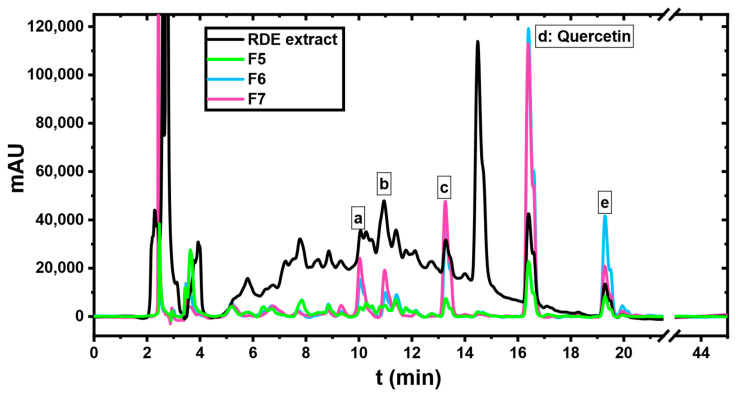
Chromatograms of RDE and fractions 5, 6 and 7 (abbreviated F5, F6 and F7).

**Figure 9:**
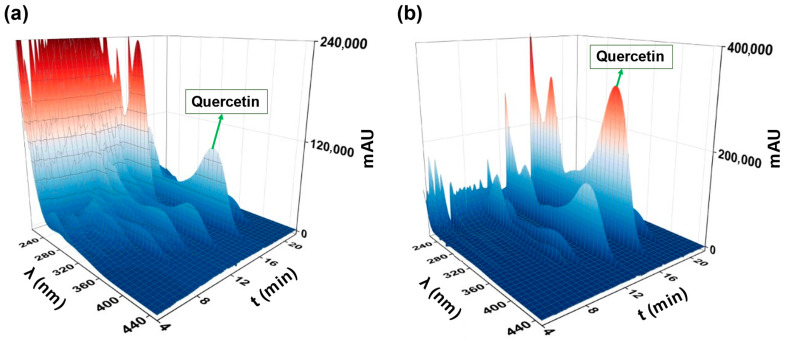
Three-dimensional chromatograms: (**a**) RDE extract; (**b**) fraction 7.

As Figure 7 shows, the peak of quercetin started to appear in the fifth fraction (80/20 MeOH/Water) and intensified through the sixth fraction (100% MeOH) until reaching the seventh one (90/10 MeOH/AcOH). The latter contained other compounds of a similar structure to quercetin such as myricetin-O-hexoside and its aglycone, quercetin-o-hexuronoside and kaempferol. To further take a comprehensive look at the fractions containing quercetin, the chromatograms of fractions 5, 6 and 7 were jointly plotted with the one of the RDE extract as shown in Figure 8. Additionally, the 3D chromatograms of the RDE extract and fraction 7 are presented in Figure 9.

**Figure 10:**
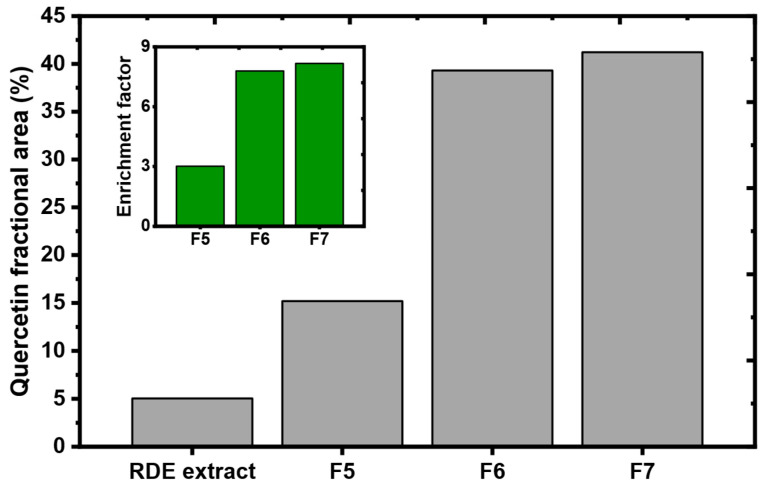
Fractional area of quercetin in RDE extract and consequent fractions (5, 6 and 7). Inset: Enrichment factors of quercetin in the last three fractions.

To acquire a clearer idea about the quercetin enrichment efficiency that occurred during the fractionation, we propose herein a simple formula that allows the calculation of the enrichment factor:

(1) $$E_{C}^{i}=\left(A_{C}^{i}/A_{tot}^{i}\right)/\left(A_{C}^{extract}/A_{tot}^{extract}\right),$$

where $E_{C}^{i}$ is the enrichment factor of a compound C in fraction *i*, $A_{C}^{i}$ is the area of the compound C in fraction *i*, $A_{tot}^{i}$ is the total area of the fraction’s chromatogram, $A_{C}^{extract}$ is the area of the compound C in the chromatogram of the RDE extract, and $A_{tot}^{extract}$ is the whole area of the extract’s chromatogram (for all the calculated areas, the system peak that appears around 2.5 min was omitted). The term $A_{C}^{i}/A_{tot}^{i}$ refers to the fractional area of a compound C in a fraction *i*. The application of this formula on fraction 5, 6 and 7 allowed obtaining the comparative bar graph for quercetin shown in Figure 10.

Since quercetin is a relatively hydrophobic molecule with a solubility ranging from 0.00215 g/L at 25 °C to 0.665 g/L at 140 °C in water [32], it started to elute in the fifth fraction where the MeOH volume concentration was 80%. The fractional area of quercetin in the sixth and seventh fractions was even higher due to the nature of the eluting solvents (100% MeOH and 90/10 MeOH/AcOH, respectively). As a result, the enrichment factors obtained increased as the water content of the eluting solvent decreased giving enrichment factors of 3, 7.5 and eventually 8. These results ascertain the capacity of MQ3 as an enriching adsorbent.

**Figure 11:**
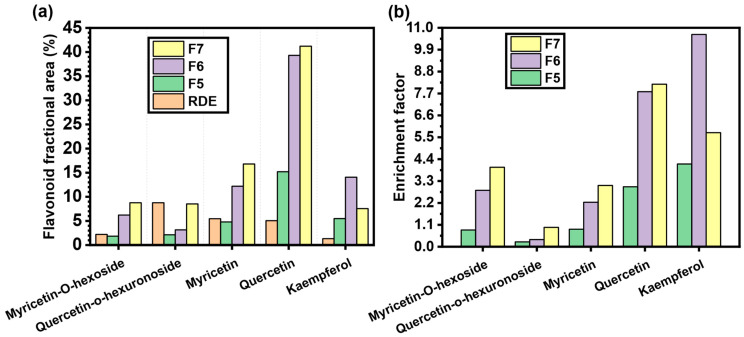
(**a**) Fractional area of each flavonoid identified in RDE and fractions 5, 6 and 7. (**b**) corresponding enrichment factors of these compounds.

It is also critical to highlight the fact that the quercetin-enriched fractions contain other flavonoids that were selectively adsorbed by the MQ3 material due to their structural similarity to quercetin. The fractional areas as well as the enrichment factors of these flavonoids were also calculated and are summarized in Figure 11. In fraction six, kaempferol was enriched the most by a factor of 10.6, whereas the enrichment of quercetin, myricetin and their glycosides was the highest in the seventh fraction. This discrepancy of enrichment factors can be attributed to the material affinity to each flavonoid, which is advantageous from a practical point of view if one desires to integrate the MIP in a continuous process of adsorption/desorption as demonstrated in the team’s previous works [30,31].

Moreover, these fractions (5, 6 and 7) can potentially have various end applications, especially in the food, pharmaceutical and cosmetic industries, due to the abundance of these fractions in flavonoids with a potential synergistic bioactivity.

## 3. Materials and Methods

### 3.1. Chemicals and Reagents

Quercetin (hydrate, 95%) and ferulic acid (99%) were both acquired form Acros Organics (Spain), while polydatin (95%) was acquired from Sigma Aldrich (Portugal). These phenolic compounds were used in the adsorption experiments to assess the synthesized MIPs.

4-vinylaniline (4VAN, 97%) and 3,5-bis(trifluoromethyl)phenyl isocyanate (BTPI, 98%) were purchased, respectively, from Sigma Aldrich (USA) and Acros Organics (USA). These two reagents were used as precursors to synthesize the 4VAN-BTPI monomer.

The monomer 4-vinylpyridine (4VP, 95%) was purchased from Alfa Aesar (USA). The styrene monomer (STY, 99%) and the crosslinker ethylene glycol dimethacrylate (EGDMA, 98%) were acquired from Sigma Aldrich (Germany). The polymerization initiator 2,2′-Azobis(2-methylpropionitrile) (AIBN, 98%) was purchased from Sigma Aldrich (Germany). The solvent dimethylformamide (DMF, 99%) was purchased from Acros Organics (Belgium). Ethanol (EtOH, 99.8%), methanol (MeOH, 99.8%), acetonitrile (ACN, 99.9%), tetrahydrofuran (THF, 99.99%) and acetic acid glacial (AcOH, 99.7%) were all acquired from Fisher chemical (UK). 

Silica gel 60 (SiO_2_, 40–63 micros) was purchased from PanReac-AppliChem (Germany). 4VP-based resins of Reillex^®^ 425 (75% of 4VP crosslinked with divinylbenzene) and Reillex^®^ 402 (98% of 4VP crosslinked with divinylbenzene) were purchased from Vertellus (USA) and Sigma-Aldrich (Portugal), respectively. Amberlite^®^ XAD4 (hydrophobic polyaromatic resin based on STY–divinylbenzene), Amberlite^®^ XAD7HP (moderately polar acrylic ester resin) and polymethylmethacrylate resin Supelite™ DAX8, an adsorbent of moderate polarity, were purchased from Sigma-Aldrich (Germany). These different resins were used in quercetin retention assays to perform comparative studies relative to the performance of the MIPs prepared in this work.

### 3.2. Synthesis of 4VAN-BTPI Monomer

The synthesis of the urea FM followed the previously reported method of Didaskalou et al. [27] and Hall et al. [33].

In a round-bottom flask and under stirring, 361.3 mg of 4VAN (3 mmol) was added to 7.5 mL of THF that was previously dried with a 3 Å molecular sieve for 80 h. The stirred solution was under an inert atmosphere of argon. Consequently, 518.5 µL of BTPI (3 mmol) was added to the mixture through a septum inserted through a rubber stopper. The reaction mixture was then left to react overnight at room temperature and under stirring. The solvent was evaporated under vacuum and the remaining solid was recrystallized from ethanol. Figure S2 shows the reaction mechanism leading to the 4VAN-BTPI monomer as well as its physical aspect. The yield was 51.8%.

### 3.3. Synthesis of Quercetin-Imprinted Polymers

To synthesize the MIPs, quercetin was first dissolved in a mixture of ACN/DMF. Then, 4VP was added and the mixture was left in an ultrasound bath for 15 min to promote the intermolecular interactions between the two. After this, STY was added to the reaction mixture and the same step of the ultrasound bath was repeated. Following this, the crosslinker EGDMA was added. Consequently, the initiator AIBN was added to the reaction mixture which was degassed for 5 min using argon to prevent AIBN cleavage by oxygen. The well-sealed glass flask was then put in a paraffin bath that had a controlled temperature of 60 °C and left to react for 24 h. This procedure was duplicated for the NIP while only omitting the quercetin. The obtained materials were washed with MeOH/AcOH 90/10 (*v*/*v*) several times then with methanol to remove the unreacted reagents as well the template from the MIP. Gravimetric analysis was used to determine the yield of the polymerization of the MIP and the NIP. 

### 3.4. FTIR

FTIR was used to analyze the synthesized 4VAN-BTPI monomer as well as the MIPs and NIPs after being washed and dried under vacuum. The apparatus used was a PerkinElmer Spectrum Two, and the characterization was carried out in attenuated total reflectance (ATR) mode between 4000 cm^−1^ and 450 cm^−1^.

### 3.5. Batch Adsorption of Polyphenols 

The synthesized materials as well as commercial resins Reillex 425, Reillex 405, DAX8, XAD4, XADHP7, and SiO_2_ were all accurately weighed (50 mg) and packed into SPE cartridges making it practical to clean the materials before and after the batch experiment as well as avoiding suspended solid in the liquid phase. Each material was conditioned by EtOH/water 80/20 (*v*/*v*) and then 1 mL of a quercetin solution (0.2 mM in EtOH/water 80/20) was loaded into the SPE cartridge. The latter was sealed both from the top and the bottom and left for 24 h in a shaker at 80 RPM. After this, the cartridges were placed in the SPE apparatus, and the liquid phases were recuperated and read in UV at 375 nm.

For MQ3 and NQ3, another set of batch experiments was made using two other phenolic compounds, ferulic acid and polydatin, to assess the selectivity of MQ3 towards quercetin.

The adsorbed amount *Q* was calculated using the following equation:

(2) $$Q=\frac{\left(C_{0}-C_{E}\right)V}{m},$$

where *C*_0_ (mM) and *C_E_* (mM) are the initial and equilibrium concentration of the phenolic compound, respectively. *V* (mL) is the volume of the testing solution and *m* (mg) is the mass of the adsorbent. Batch results for the different materials and phenolic compounds are presented in Tables S1–S3 in the Supplementary Material.

### 3.6. Extraction of Diatomaceous Earth

RDE was retrieved from the winery of Caves Campelo in the region of Barcelos-Braga in Portugal. The RDE was used as received to perform the extraction. Briefly, 500 mL of ethanol/water 80/20 was mixed with 250 g of RDE (ratio of 50 g/100 mL) in a beaker and then left in an ultrasound bath for 30 min. Consequently, the beaker was left for 2 h under stirring and wrapped in aluminum to prevent the photodegradation of phenolic compounds. At the end of the two hours, the mixture was filtered into a Büchner flask resulting in a dark-red liquid extract exempt of solid residues. 

### 3.7. RDE Extract Treatment by a MIP

Before the saturation, the MQ3 material was thoroughly cleaned and conditioned with the same solvent of the RDE extract (80/20 EtOH/water). After that, and by means of SPE set-up, the extract was passed through the material 1 mL at a time while monitoring the UV response of the cartridge’s outlet until reaching the saturation. Following this, the process of fractionation took place using the following sequence:

1. 100% Water: F1;
2. Water/MeOH 80/20 (*v*/*v*): F2;
3. Water/MeOH 60/40 (*v*/*v*): F3;
4. Water/MeOH 40/60 (*v*/*v*): F4;
5. Water/MeOH 20/80 (*v*/*v*): F5;
6. 100% MeOH F6;
7. MeOH/AcOH 90/10 (*v*/*v*): F7.

Each fraction was obtained by eluting 1 mL of the mentioned solvents.

### 3.8. UHPLC-DAD Analysis 

The fractions obtained from the MQ3 material as well the saturating RDE extract were analyzed with an Ultra-High-performance Liquid Chromatography coupled to Diode-Array Ultraviolet Detector (UHPLC-DAD). The latter was supplied by KNAUER and consisted of a P6.1 L gradient pump equipped with a degasser, a 6.1 L autosampler, a column thermostat CT2.1 and a diode array detector 6.1 L. KNAUER ClarityChrom^®^ was the software that allowed the control of the UHPLC-DAD system. The chromatographic analysis was performed using an Ascentis^®^ C18 (SUPELCO^®^) column with a particle size of 5 µm and dimensions 25 cm × 4.6 mm. A gradient of solvents was used as a mobile phase varying from A 100% (90/10 water/ACN) to B (10/90 water/ACN) 100% for 45 min. In both solvents A and B, the water’s pH was set to 3 using AcOH. The flow rate was 1 mL/min, and the temperature of the column was fixed at 45 °C. Additionally, the injection volume of the samples was 50 µL. The acquired chromatograms are plotted at 280 nm.

Since the purpose of this work is to demonstrate the enrichment of quercetin by means of the synthesized MIPs, the standard of quercetin was injected using the same method of the samples, which allowed a direct comparison of the retention time and the UV-vis spectrum of quercetin. Nevertheless, a tentative identification of other phenolic compounds was carried out relying on the LC-MS-DAD data of the RDE reported by fellow researchers of CIMO. The LC-MS-DAD/UHPLC-DAD comparison relied mainly on the UV-vis spectra of the compounds as well as their elution order. The conditions of LC-MS-DAD are detailed elsewhere [34].

## 4. Conclusions

The present paper was dedicated to the valorization of phenolic compounds from residual diatomaceous earth, which is often an overlooked winemaking residue. To this end, tailor-made adsorbents were synthesized through free radical polymerization using monomers that rely on hydrogen bonding and π-π stacking as binding mechanisms. The chosen template for the molecular imprinting was quercetin which is a typical flavonoid encountered in wine and winemaking residues. 

During the synthesis step, different ratios of monomers and crosslinker were used to assess their influence on the MIP’s performance. Batch sorption was used to measure the retention capability of each MIP (and its corresponding NIP) considering individual assays with quercetin, ferulic acid and polydatin. Among the synthesized MIPs, the one containing the lowest amount of crosslinker (Y_CL_ = 40%) and possessing microparticles (due to Y_M_ = 6%) demonstrated the highest retention for quercetin as well as imprinting effect towards the same compound. 

This 4VP-based MIP was denominated as MQ3 and was utilized to retrieve polyphenols contained in the RDE extract in a process of adsorption followed by desorption. The aptitude of MQ3 to selectively retain quercetin was proved through the chromatographic analyses of the desorbed fractions which showed an eightfold increase in quercetin concentration (compared to the original RDE extract). Besides quercetin, other flavonoids such as myricetin and its glycosylated form, kaempferol and quercetin-o-hexuronoside were also enriched using the synthesized MIP. This result is interesting since a rich fraction of flavonoids could be useful in the pharmaceutical or the cosmetic industry. 

In spite of the auspicious results obtained herein with the synthesized MIP (MQ3), further studies regarding the design of MIPs are recommended to enhance the molecular-imprinting process which should translate into a better MIP selectivity. These studies should focus on varying the ratios of crosslinker and monomers to an even further degree and experimenting with other templates (instead of quercetin) to assess the role of the imprinting molecule on the enrichment process of polyphenols. Furthermore, as promising as MIP technology seems to be, scaling up the synthesis for mass production could pose some cost problems due to the price of the utilized reagents.

All in all, through the present work outputs, the usefulness of MIPs in generating added value to winemaking residues is demonstrated, reinforcing the path towards a circular bioeconomy.

## Figures and Tables

**Table 1:** Polymerization conditions used in the preparation of different MIPs.

Material	Template	FM1	FM2	Solvent (*v*/*v*)	Y_M_	Y_I_	Y_CL_	Y_FM1/T_	Y_FM2/T_	Yield (%)	Comments
MQ1 NQ1	Quercetin N/A	4VAN-BTPI	STY	ACN/DMF 85/15	23.97	5.32	83.33	2 N/A	1 N/A	81.73 86.70	Bulky material
MQ2	Quercetin	4VP	STY	ACN/DMF 85/15	21.46	5.32	83.33	2	1	95.9	Bulky material
NQ2	N/A							N/A	N/A	97.9	
MQ3	Quercetin	4VP	STY	ACN/DMF 85/15	6.00	5.22	40	2	1	68.6	Particle powder
NQ3	N/A							N/A	N/A	74.3	
MQ4	Quercetin	4VP	STY	ACN/DMF 70/30	21.76	5.33	40	2	1	96.1	Bulky material
NQ4	N/A							N/A	N/A	96.8	

For all the materials, free radical polymerization was performed at 60 °C, using AIBN as initiator and ethylene glycol dimethacrylate (EGDMA) as crosslinker.

**Table 2:** Tentative identification of the phenolic compounds in the hydroethanolic extract of RDE.

Peak	Retention Time (min)	Tentative Identification
a	10.06	Myricetin-O-hexoside
b	10.9	Quercetin-o-hexuronoside
c	13.3	Myricetin
d	16.4	Quercetin
e	19.3	Kaempferol

## Data Availability

Not applicable.

## Supplementary Materials

The following supporting information can be downloaded at: https://www.mdpi.com/article/10.3390/molecules27196406/s1, Figure S1: FTIR spectra of MQ4 and NQ4; Figure S2: (a) Reaction mechanism of the 4VAN-BTPI monomer; (b) Photograph of the synthesized urea monomer; Table S1: Batch results for quercetin 0.2 mM in 8/2 EtOH/Water; Table S2: Batch results for ferulic acid 0.2 mM in 8/2 EtOH/Water; Table S3: Batch results for polydatin 0.2 mM in 8/2 EtOH/Water.

## Sample Availability

Not available.
