# Supplementary material for: Enrichment of Quercetin from Winemaking Residual Diatomaceous Earth via a Tailor-Made Imprinted Adsorbent
Source: Molecules. 2022 Sep 28;27(19):6406. doi: 10.3390/molecules27196406 (PMC9570924; doi:10.3390/molecules27196406)
Supplement: Supplementary file 1 [file molecules-27-06406-s001.zip › molecules-1862449-supplementary.pdf]

molecules

MDPI

Supplementary material

Enrichment of Quercetin from Winemaking Residual Diatomaceous Earth via a Tailor-Made Imprinted Adsorbent

Amir Bzainia, Rolando C.S. Dias and Mário Rui P.F.N. Costa

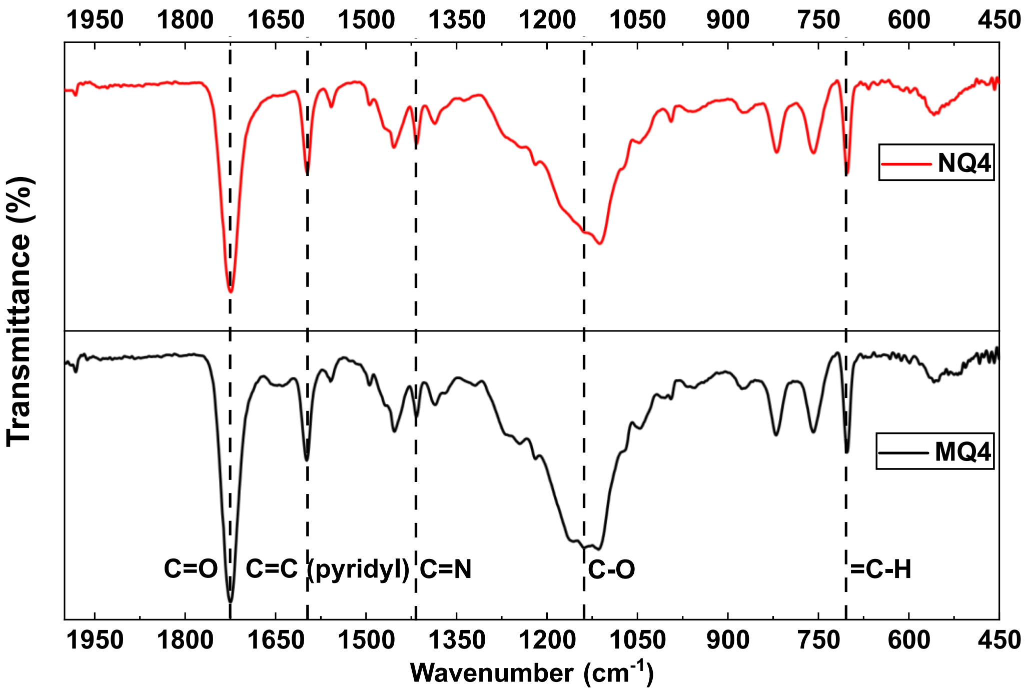

Figure S1. FTIR spectra of MQ4 and NQ4

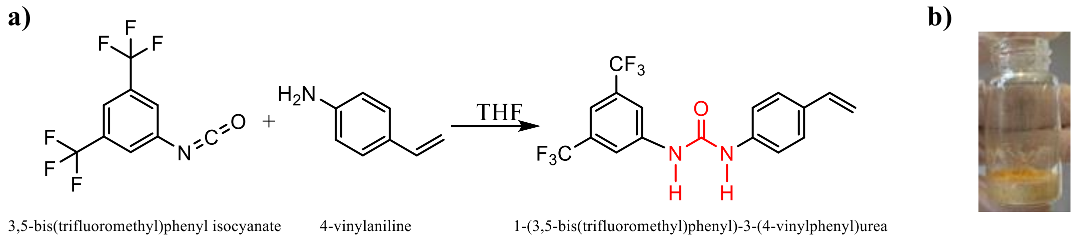

Figure S2. a) Reaction mechanism of the 4VAN-BTPI monomer; **b)** Photograph of the synthesized urea monomer.

Table S1. Batch results for quercetin 0.2mM in 8/2 EtOH/Water

Material	MQ1	NQ1	MQ2	NQ2	MQ3	NQ3	MQ4	NQ4	Reillex 425	Reillex 402	DAX8	XAD4	XADHP7	SiO_2
Q (µmol/g) 1st assay	1.180	0.948	2.697	1.886	3.956	3.250	3.485	3.500	2.839	3.901	0.984	0.683	1.084	0.695
Q (µmol/g) 2nd assay	1.136	0.767	2.445	2.005	3.362	2.910	2.837	3.091	2.41940	3.071	0.749	0.751	0.708	0.570
Q (µmol/g) 3rd assay	1.274	1.073	2.824	2.274	3.385	2.891	3.227	3.413	2.368	3.159	0.757	0.673	0.717	0.618
Q (µmol/g) Average	1.197	0.929	2.655	2.055	3.568	3.017	3.183	3.335	2.542	3.377	0.830	0.702	0.836	0.628
Standard deviation	0.057	0.125	0.157	0.162	0.274	0.165	0.266	0.175	0.211	0.372	0.108	0.034	0.175	0.051

Table S2. Batch results for ferulic acid 0.2mM in 8/2 EtOH/Water

Material	MQ3	NQ3
Q (µmol/g) 1st assay	2.537	1.491
Q (µmol/g) 2nd assay	2.246	1.297
Q (µmol/g) 3rd assay	2.234	1.817
Q (µmol/g) Average	2.339	1.535
Standard deviation	0.140	0.214

Table S3. Batch results for polydatin 0.2mM in 8/2 EtOH/Water

Material	MQ3	NQ3
Q (µmol/g) 1st assay	2.794	2.597
Q (µmol/g) 2nd assay	2.238	1.969
Q (µmol/g) 3rd assay	2.903	2.730

Q (μmol/g) Average	2.645	2.432
Standard deviation	0.291	0.332
